# Temporal trends in the prevalence and incidence of depression and the interplay of comorbidities in patients with young- and usual-onset type 2 diabetes from the USA and the UK

**DOI:** 10.1007/s00125-022-05764-9

**Published:** 2022-09-05

**Authors:** John Dibato, Olga Montvida, Joanna Ling, Digsu Koye, William H. Polonsky, Sanjoy K. Paul

**Affiliations:** 1 Melbourne EpiCentre, Department of Medicine at Royal Melbourne Hospital, University of Melbourne, Melbourne, VIC Australia; 2 Department of Family and Community Medicine, University of California, San Diego, CA USA; 3 Present Address: AstraZeneca, London, UK

**Keywords:** Antidepressants, Depression, Diabetes, Mental illness, Real-world evidence

## Abstract

**Aims/hypothesis:**

We aimed to investigate the prevalence and incidence of depression, and the interplay of cardiometabolic comorbidities, in the differentiation of depression risk between young-onset diabetes (diagnosis at age <40 years) and usual-onset diabetes (diagnosis at age ≥40 years).

**Methods:**

Using electronic medical records from the UK and USA, retrospective cohorts of adults with incident type 2 diabetes diagnosed between 2006 and 2017 were examined. Trends in the prevalence and incidence of depression, and risk of developing depression, in participants with young-onset type 2 diabetes compared with usual-onset type 2 diabetes were assessed separately by sex and comorbidity status.

**Results:**

In total 230,932/1,143,122 people with type 2 diabetes from the UK/USA (mean age 58/60 years, proportion of men 57%/46%) were examined. The prevalence of depression in the UK/USA increased from 29% (95% CI 28, 30)/22% (95% CI 21, 23) in 2006 to 43% (95% CI 42, 44)/29% (95% CI 28, 29) in 2017, with the prevalence being similar across all age groups. A similar increasing trend was observed for incidence rates. In the UK, compared with people aged ≥50 years with or without comorbidity, 18–39-year-old men and women had 23–57% and 20–55% significantly higher risks of depression, respectively. In the USA, compared with those aged ≥60 years with or without comorbidity, 18–39-year-old men and women had 5–17% and 8–37% significantly higher risks of depression, respectively.

**Conclusions/interpretation:**

Depression risk has been increasing in people with incident type 2 diabetes in the UK and USA, particularly among those with young-onset type 2 diabetes, irrespective of other comorbidities. This suggests that proactive mental health assessment from the time of type 2 diabetes diagnosis in primary care is essential for effective clinical management of people with type 2 diabetes.

**Graphical abstract:**

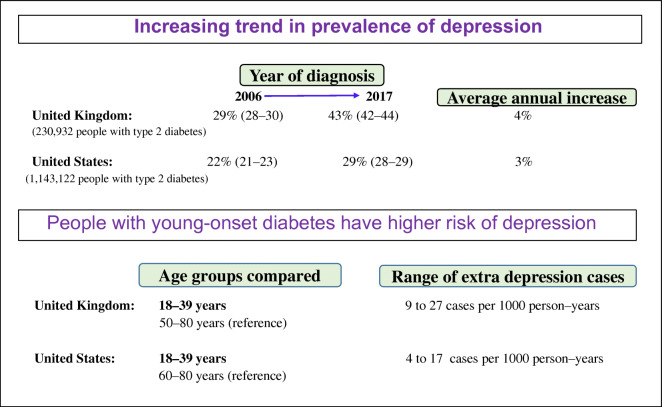

**Supplementary Information:**

The online version contains peer-reviewed and unedited supplementary material available at 10.1007/s00125-022-05764-9.

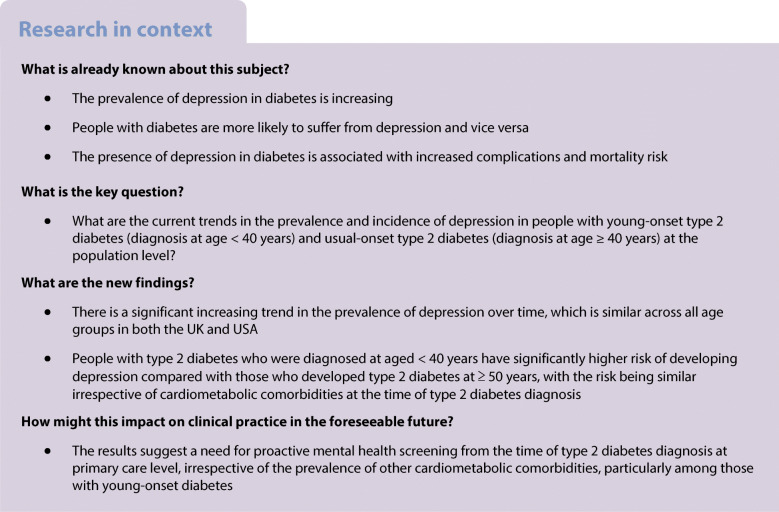



## Introduction

Depression and diabetes are complex disease conditions that commonly co-occur [1]. People with type 2 diabetes are at higher risk of presenting with depressive symptoms compared with those without type 2 diabetes [1, 2]. Meta-analyses suggest that the global prevalence of depression in type 2 diabetes has risen from 20% (results published in 2007) to 32%, based on studies published in 2018, and diabetes is associated with a 36–64% increased risk of developing depression [3, 4]. The co-occurrence of depression and type 2 diabetes is associated with poorer adherence to medical treatment, lower quality of life and increased risk of developing long-term microvascular and macrovascular complications, as well as increased mortality risk [5, 6].

While the connection between type 2 diabetes and depression is well-known, the specific burdens of depression in young-onset type 2 diabetes (YOD, diagnosis at age <40 years) and usual-onset type 2 diabetes (diagnosis at age ≥40 years) have received little attention. Recent studies suggest that there is an increasing trend for YOD in many countries, particularly in the USA and the UK, with YOD having more of an aggressive disease phenotype, leading to premature development of complications [5–9]. People with YOD are more likely to develop mental illnesses and microvascular and macrovascular complications as they age [5–7, 10], with higher rates of hospitalisations.

Recent studies addressing the diabetes/depression association are primarily based on cross-sectional data, with little longitudinal population-level data addressing the presumed temporal associations [2, 11]. Furthermore, while some studies have reported that depression is less common in the older type 2 diabetes population (>60 years old) [12], other studies have contradicted these findings [13, 14]. The relationship between age and risk of depression among people with type 2 diabetes is inherently complex and requires further investigation to support effective primary care-led chronic disease management and reduction in health cost. To the best of our knowledge, no study has yet examined the temporal trends for depression at type 2 diabetes onset using population-level data. While YOD has been shown to be associated with an increased risk of major chronic conditions [7, 15], its interaction with other comorbidities in men and women, and the impact of such interaction on the risk of developing depression in people with incident type 2 diabetes, have not been studied. Previous studies have shown and discussed fundamental differences in people with and without cardiometabolic comorbidities at type 2 diabetes diagnosis [5, 9]. The various combinations of comorbidities present before and after diagnosis of diabetes may drive different levels of risk between different age groups and between men and women. Finally, while recent longitudinal studies based on electronic medical records (EMRs) have reported an increasing prevalence of YOD in the USA and the UK, which have different healthcare systems [5, 8, 9], it is of great importance to also evaluate whether the risk dynamics of depression along with other comorbidities in people with a new diagnosis of type 2 diabetes are similar at the population level in these different healthcare systems.

Using nationally representative EMRs from the UK and USA for people with incident type 2 diabetes, the aims of this study were to: (1) explore temporal trends in the prevalence and incidence of depression in people with young- and usual-onset type 2 diabetes by sex; (2) examine the risk of developing depression in people with YOD compared with those with older-onset type 2 diabetes by sex; and (3) evaluate the effect of the interaction between YOD and comorbidities at type 2 diabetes diagnosis on the risk of developing depression.

## Methods

This study was performed according to the Reporting of Studies Conducted Using Observational Routinely Collected Data (RECORD) guidelines (http://www.record-statement.org).

### Data

Two nationally representative EMRs were used in this study: the Health Improvement Network (THIN), which represents over 770 primary care practices across the UK, and Centricity Electronic Medical Records (CEMR), which represents over 40 000 ambulatory and primary care medical practices from across all states in the USA. These databases are generally representative of the UK and US populations with respect to demographics, major disease prevalence and cardiometabolic risk factors [16, 17]. Longitudinal records were available from 2006 to 2017 for more than 17/46 million individuals from THIN/CEMR, with comprehensive patient-level information on demographic and anthropometric variables, clinical and laboratory measures, prescription drugs and disease events. All disease diagnoses were coded according to the Read codes (https://digital.nhs.uk/article/1104/Read-Codes) in the 9th and 10th revisions to the International Classification of Diseases (ICD-9 and ICD-10) (http://www.icd9data.com/2007/Volume1/default.htm; http://apps.who.int/classifications/icd10/browse/2016/en).

### Study cohort identification

The study cohorts from the UK and US databases were identified using the following conditions: (1) data available for age and sex, (2) aged 18–80 years at the time of type 2 diabetes diagnosis; (3) diagnosed on or after 1 January 2006 to 30 September 2017; and (4) date of type 2 diabetes diagnosis at least 6 months after registering into the EMRs to reduce bias in identifying incident cases. The clinically guided machine learning-based algorithms to identify patients with type 2 diabetes from EMRs have been described previously [18, 19]. The date of type 2 diabetes diagnosis was defined as the index date.

### Study variables

For both cohorts of people with incident type 2 diabetes, data on prescriptions, disease diagnoses, demographic variables, and clinical and laboratory measurements were extracted. Details of the methodology used for data extraction and assessment of longitudinal patient-level risk factors, disease events and medication data from THIN and CEMR databases have been described previously [5, 19–22]. Baseline demographic variables and clinical and laboratory measures included age at type 2 diabetes diagnosis (18–39, 40–49, 50–59, 60–69 and 70–79 years), sex, ethnicity (USA only), Townsend deprivation score (UK only), smoking status, BMI, HbA_1c_, and systolic BP. Young-onset diabetes included patients diagnosed with type 2 diabetes at age <40 years while usual-onset diabetes included patients diagnosed at age ≥40 years.

Any mental illness (AMI) was defined as the presence of (1) mental illness that meet diagnostic criteria specified within DSM-5, excluding developmental and substance use disorders [23], or (2) at least two prescriptions for antidepressant medication. AMI included depression, anxiety, bipolar disorder, schizophrenia, post-traumatic stress disorder, eating disorder, gender dysphoria, personality disorder and other unspecified mental illnesses. Depression was defined using a clinically guided machine learning-based algorithm [24, 25]. The definition included those with a diagnostic code or at least two prescriptions (within a 6-month window) for antidepressants used for treating depression: antidepressant medications were limited to those commonly prescribed for depression (electronic supplementary material [ESM] Tables 1 and 2). The algorithm accounted for other mental illnesses specified within AMI, and all diagnoses of depression or AMI were based on first occurrences of the disease during the study period. Atherosclerotic cardiovascular disease was identified as a clinical diagnosis of myocardial infarction, ischaemic heart disease, unstable angina, ischaemic stroke, haemorrhagic stroke, transient ischaemic attack, peripheral vascular disease or cerebrovascular disease. The definition of any cardiovascular disease included atherosclerotic cardiovascular disease and heart failure. Chronic kidney disease included diagnostic codes for stages 2–5 or end-stage renal disease, nephropathy and kidney dialysis, or a GFR <60 ml/min per 1.73 m^2^ or a urine albumin/creatinine ratio >30 mg/mmol (300 mg/g). Microvascular disease was defined as a clinical diagnosis of neuropathy, retinopathy or chronic kidney disease. Cancer included any malignant neoplasm excluding skin cancer. Hypertension/dyslipidaemia were defined as the presence of a clinical diagnosis or use of antihypertensive/lipid-lowering drugs. Comorbidities included any cardiovascular disease, microvascular diseases, obesity grade 2 + (BMI ≥35 kg/m^2^) or cancer. A disease was considered as prevalent if its first available diagnostic date was on or prior to the index date, and incident if the first diagnosis occurred after the index date.

### Ethics statement

The protocol for the UK data was approved by the Scientific Review Committee of IQVIA Medical Research Data UK, incorporating THIN (protocol number SRC Protocol 19THIN081-v1-11-102019). This study also involved the use of EMRs from the USA in which patients could not be identified directly or through identifiers linked to them. According to US Department of Health and Human Services Exemption 4 (CFR 46.101(b)(4)), this study is exempt from ethics approval from an institutional review board and informed consent.

### Statistical analysis

Baseline characteristics are summarised by number (%), mean ± SD or median (IQR) as appropriate by age groups. Results are reported in the order UK/USA where appropriate.

The crude prevalence (95% CI) of depression was estimated by age at type 2 diabetes diagnosis, sex, and year of type 2 diabetes diagnosis. This was done by summing the number of patients with prevalent events and dividing by the total number at index date. Among patients without history of AMI at the index date, we estimated the crude incidence rates of depression per 1000 person-years (PTPY; 95% CI) by age at type 2 diabetes diagnosis, sex, baseline comorbidities and year at type 2 diabetes diagnosis. The follow-up time was measured from type 2 diabetes diagnosis date to the date of occurrence of depression or the end of the study follow-up if depression did not occur.

To evaluate the changing patterns in individual trends for prevalence and incidence of depression, the joinpoint regression based on annual percentage change estimates was obtained [26]. Among people without AMI at type 2 diabetes diagnosis, survival models were used to evaluate the risk of developing depression in people aged 18–39 years compared with older age groups, separately in men and women and by comorbidity status [27, 28]. The HRs and 95% CIs were obtained in addition to absolute risk (AR, additional number of depression cases PTPY) in the youngest age group compared with the older age groups.

Several survival models were assessed using Akaike’s information criteria, from which the Weibull model was chosen as the best fit for estimating the HR, while Aalen’s additive hazards model was used to estimate the AR of depression. The baseline survival model included age at diagnosis, deprivation status (UK only), smoking status, ethnicity (USA only), hypertension and dyslipidaemia.. Based on Akaike’s information criteria, dyslipidaemia was dropped from the final model. Missing data for ethnicity, deprivation and smoking status were included as a categorical field (missing or unknown).

## Results

### Patient characteristics at type 2 diabetes diagnosis 

A total of 230,932/1,143,122 people diagnosed with type 2 diabetes in the UK/USA met the inclusion criteria (ESM Fig. 1), with a mean follow-up of 5.0/4.6 years (Table 1). At baseline, the mean age (±SD) was 58 ± 13/60 ± 13 years, with 57%/46% men, 21%/21% current smokers, 61%/73% with hypertension, 48%/33% with AMI and 53%/62% with one or more comorbidity (Tables 1 and 2). Obesity was significantly higher among young adults (71%/77% in those aged 18–39 years at diagnosis, and 72%/79% in those aged 40–49 years), compared with older age groups. The mean HbA_1c_ and proportion with HbA_1c_ ≥59 mmol/mol (7.5%) among the group diagnosed at 18–39 years old (70 mmol/mol [8.6%]/64 mmol/mol [8.0%] and 58%/45%, respectively) were significantly higher compared with those diagnosed at 40–49 years old. The prevalence of comorbidities was highest in the group aged 70–79 years (70%/65%), and that in the group aged 18–39 years were 36%/59%. In the UK, younger adults were most deprived compared with older adults, and in the USA, the distributions of ethnicity were similar across age groups (ESM Tables 3 and 4).

**Table 1:** Patients’ demographic and clinical characteristics at the time of type 2 diabetes diagnosis by age groups in the UK and US cohorts

Variable	Age at type 2 diabetes diagnosis (years)										Overall	
	18–39		40–49		50–59		60–69		70–79			
	UK	USA	UK	USA	UK	USA	UK	USA	UK	USA	UK	USA
*n* (%)	18,809 (8)	84,851 (7)	37,157 (16)	146,953 (13)	59,105 (26)	281,957 (25)	66,573 (29)	321,114 (28)	49,288 (21)	308,247 (27)	230,932	1,143,122
Follow-up (years)												
Mean ± SD	5.0 ± 3.1	4.4 ± 3.2	5.1 ± 3.1	4.6 ± 3.2	5.1 ± 3.1	4.6 ± 3.2	5.0 ± 3.1	4.6 ± 3.2	4.7 ± 3.0	4.4 ± 3.1	5.0 ± 3.1	4.6 ± 3.2
Age (years)												
Mean ± SD	33 ± 5	33 ± 5	45 ± 3	45 ± 3	55 ± 3	55 ± 3	64 ± 3	65 ± 3	74 ± 3	74 ± 3	58 ± 13	60 ± 13
Men	9381 (50)	28,000 (33)	22,904 (62)	64,431 (44)	35,447 (60)	128,697 (46)	38,637 (58)	153,445 (48)	25,009 (51)	146,371 (47)	131,378 (57)	520,944 (46)
Smoking status												
Non-missing	18,658 (99)	56,686 (67)	36,981 (100)	98,093 (67)	58,858 (100)	188,523 (67)	66,146 (99)	209,193 (65)	48,692 (99)	185,399 (60)	229,335 (99)	737,894 (65)
Current smoker	5661 (30)	22,611 (27)	10,366 (28)	40,331 (27)	14,143 (24)	74,407 (26)	12,164 (18)	68,025 (21)	5408 (11)	39,560 (13)	47,742 (21)	244,934 (21)
Weight (kg)												
Non-missing	12,642 (67)	76,399 (90)	27,461 (74)	128,070 (87)	44,283 (75)	239,314 (85)	50,093 (75)	267,992 (83)	35,910 (73)	248,714 (81)	170,389 (74)	960,489 (84)
Mean ± SD	102 ± 27	106 ± 31	101 ± 24	105 ± 27	97 ± 21	100 ± 24	91 ± 19	95 ± 22	84 ± 17	85 ± 20	93 ± 22	96 ± 25
BMI (kg/m^2^)												
Non-missing	12,513 (67)	75,446 (89)	27,264 (73)	126,468 (86)	44,032 (74)	236,304 (84)	49,854 (75)	264,012 (82)	35,667 (72)	242,208 (79)	169,330 (73)	944,438 (83)
Mean ± SD	35 ± 8	38 ± 10	35 ± 8	37 ± 9	34 ± 7	35 ± 8	32 ± 6	34 ± 7	30 ± 6	31 ± 7	33 ± 7	34 ± 8
Obesity grade 1	3042 (24)	15,232 (20)	7824 (29)	32,375 (26)	14,055 (32)	67,970 (29)	16,383 (33)	80,198 (30)	10,768 (30)	67,658 (28)	52,072 (31)	263,433 (28)
Obesity grade 2+	5873 (47)	43,167 (57)	11,659 (43)	67,242 (53)	15,705 (36)	106,847 (45)	13,312 (27)	95,762 (36)	6,007 (17)	50,324 (21)	52,556 (31)	363,342 (38)
Systolic BP (mmHg)												
Non-missing	13,170 (70)	76,134 (90)	29,879 (80)	127,138 (87)	49,858 (84)	235,876 (84)	58,167 (87)	263,174 (82)	43,479 (88)	246,845 (80)	194,553 (84)	949,167 (83)
Mean ± SD	130 ± 15	125 ± 14	135 ± 16	129 ± 15	138 ± 16	131 ± 15	139 ± 16	132 ± 16	139 ± 16	132 ± 17	138 ± 16	131 ± 16
Uncontrolled systolic BP	3274 (25)	10,915 (14)	11,542 (39)	27,489 (22)	24,105 (48)	65,235 (28)	32,176 (55)	89,021 (34)	25,357 (58)	93,259 (38)	96,454 (50)	285,919 (30)
HbA_1c_												
Non-missing	10,521 (56)	46,449 (55)	25,309 (68)	77,706 (53)	41,997 (71)	134,941 (48)	47,727 (72)	137,529 (43)	35,149 (71)	115,689 (38)	160,703 (70)	512,314 (45)
Mean ± SD (mmol/mol)	70 ± 26	64 ± 25	69 ± 25	61 ± 23	65 ± 24	59 ± 26	61 ± 22	56 ± 18	58 ± 20	52 ± 14	63 ± 23	57 ± 20
Mean ± SD (%)	8.6 ± 2.4	8.0 ± 2.3	8.4 ± 2.4	7.8 ± 2.1	8.1 ± 2.2	7.6 ± 2.0	7.7 ± 2.0	7.2 ± 1.7	7.5 ± 1.9	6.9 ± 1.3	7.9 ± 2.1	7.4 ± 1.8
HbA_1c_ ≥59 mmol/mol (7.5%)	6054 (58)	21,091 (45)	13,578 (54)	30,747 (40)	19,120 (46)	46,513 (34)	17,244 (36)	37,050 (27)	10,372 (30)	21,940 (19)	66,368 (41)	157,341 (31)

Data are presented as *n* (%), or means ± SD where indicated

Obesity grade 1 represents a BMI between 30 and 35 kg/m^2^; obesity grade 2+ represents a BMI of at least 35 kg/m^2^

Uncontrolled systolic BP includes individuals with ASCVD and having a systolic BP of at least 130 mmHg, or individuals without ASCVD and having a systolic BP of at least 140 mmHg

ASCVD, atherosclerosis cardiovascular disease

**Table 2:** Patients’ comorbidities at the time of type 2 diabetes diagnosis by age groups in the UK and US cohorts

Variable	Age at type 2 diabetes diagnosis (years)										Overall	
	18–39		40–49		50–59		60–69		70–79			
	UK	USA	UK	USA	UK	USA	UK	USA	UK	USA	UK	USA
*n* (%)	18,809 (8)	84,851 (7)	37,157 (16)	146,953 (13)	59,105 (26)	281,957 (25)	66,573 (29)	321,114 (28)	49,288 (21)	308,247 (27)	230,932	1,143,122
Any mental illness	8016 (43)	31,351 (37)	17,989 (48)	57,593 (39)	29,280 (50)	105,282 (37)	32,561 (49)	105,136 (33)	22,673 (46)	81,505 (26)	110,519 (48)	380,867 (33)
Depression	6063 (32)	22,300 (26)	13,902 (37)	41,940 (29)	21,891 (37)	79,277 (28)	23,081 (35)	80,868 (25)	15,993 (32)	63,557 (21)	80,930 (35)	287,942 (25)
ASCVD	282 (1)	2289 (3)	1993 (5)	9541 (6)	7095 (12)	35,341 (13)	14,506 (22)	63,381 (20)	16,012 (32)	88,177 (29)	39,888 (17)	198,729 (17)
Any cardiovascular disease	314 (2)	2914 (3)	2132 (6)	11,270 (8)	7404 (13)	39,499 (14)	15,120 (23)	69,355 (22)	16,823 (34)	97,321 (32)	41,793 (18)	220,359 (19)
Chronic kidney disease	608 (3)	4094 (5)	2534 (7)	12,782 (9)	7661 (13)	35,952 (13)	16,736 (25)	65,342 (20)	21,832 (44)	97,745 (32)	49,371 (21)	215,915 (19)
Microvascular disease	897 (5)	11,703 (14)	3304 (9)	30,694 (21)	9163 (16)	69,947 (25)	18,560 (28)	96,429 (30)	22,971 (47)	120,113 (39)	54,895 (24)	328,886 (29)
Cancer	268 (1)	1457 (2)	894 (2)	4340 (3)	2702 (5)	12,741 (5)	5844 (9)	23,284 (7)	6759 (14)	34,045 (11)	16,467 (7)	75,867 (7)
Hypertension	4154 (22)	34,839 (41)	15,296 (41)	88,576 (60)	34,248 (58)	201,483 (71)	47,404 (71)	253,567 (79)	39,787 (81)	258,058 (84)	140,889 (61)	836,523 (73)
Dyslipidaemia	1439 (8)	23,425 (28)	7450 (20)	71,091 (48)	20,745 (35)	171,362 (61)	33,887 (51)	223,374 (70)	29,410 (60)	222,991 (72)	92,931 (40)	712,243 (62)
Comorbidity	6805 (36)	49,782 (59)	15,379 (41)	88,135 (60)	27,538 (47)	168,354 (60)	37,488 (56)	198,467 (62)	34,356 (70)	201,108 (65)	121,566 (53)	705,846 (62)

Date are presented as *n* (%)

Any cardiovascular disease includes ASCVD + heart failure

Cancer cases involve all malignant cancer excluding skin cancer

Microvascular diseases include retinopathy, neuropathy and nephropathy including chronic kidney disease

Comorbidity comprises at least one of cardiovascular disease, cancer, microvascular disease and obesity grade 2+

ASCVD, atherosclerosis cardiovascular disease

### Temporal trends in the prevalence and incidence of depression

**Fig. 1:**
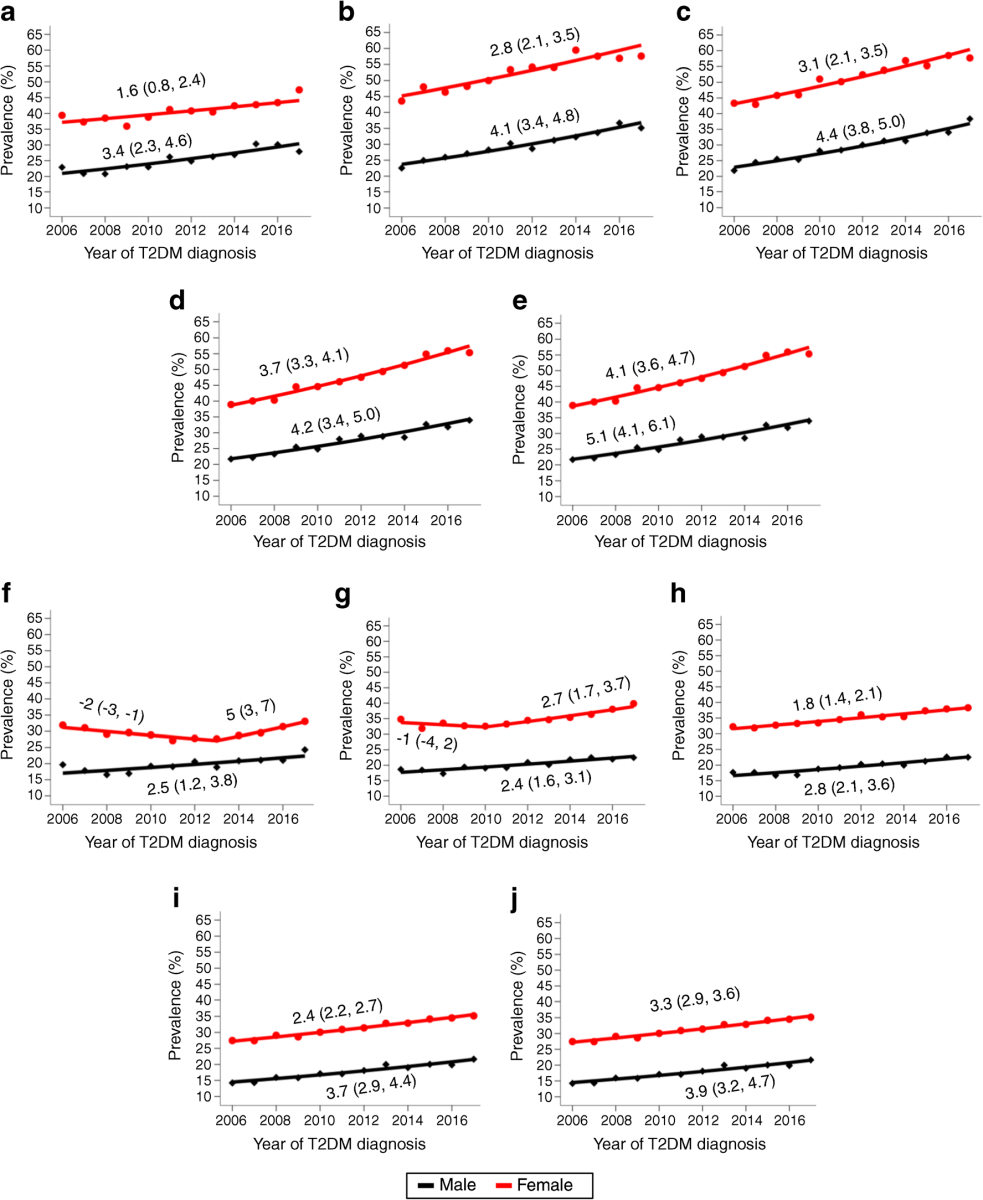
Observed and modelled trends in the prevalence of depression among incident type 2 diabetes (T2DM) patients from the UK and the USA. The trends are represented separately by age at diagnosis of type 2 diabetes and sex. (**a–e**) UK trends for the 18–39 (**a**), 40–49 (**b**), 50–59 (**c**), 60–69 (**d**) and 70–79 (**e**) year age groups; (**f–j**) US trends for the 18–39 (**f**), 40–49 (**g**), 50–59 (**h**), 60–69 (**i**) and 70–79 (**j**) year age groups. The black diamonds and red circles represent observed prevalence for men and women, respectively; the lines are estimates from the joinpoint regression model [29]. The numbers shown within the plots are the mean annual percentage change (95% CI)

The overall prevalence of AMI and depression by age groups is presented in Table 2. The depression prevalence was similar between the group aged 18–39 years (32/26%) and those aged 70–79 years (32/21%). The temporal trend in the prevalence of depression has been significantly increasing over the last 10 years across all age groups in both men and women, with women having a consistently significantly higher prevalence compared with men in both countries (Fig. 1). Overall, the prevalence of depression increased from 29% (95% CI 28, 30)/22% (95% CI 21, 23) in 2006 to 43% (95% CI 42, 44)/29% (95% CI 28, 29) in 2017 (ESM Fig. 2), with annual increasing rates of 3.8%/2.8%.

The overall incidence PTPY for depression also increased from 40 PTPY (95% CI 39, 41)/33 PTPY (95% CI 32, 34) in 2006 to 50 PTPY (95% CI 45, 55)/62 PTPY (95% CI 60, 64) in 2016 (ESM Fig. 2). Men and women in the UK aged <50 years had numerically higher incidence rates compared with those aged 50–69 years at type 2 diabetes diagnosis, with or without comorbidity at baseline (Table 3). However, the presence of a baseline comorbidity led to a significant increase in depression incidence among those aged 18–39 years at diagnosis, compared with older age groups, consistent across sex in both countries.

**Table 3:** Unadjusted rates for depression onset by age, sex and baseline comorbidity in individuals with incident type 2 diabetes from the UK and USA

Age group	Men (UK)	Women (UK)	Men (USA)	Women (USA)
Total population				
18–39 years	47.66 (45.06, 50.42)	66.42 (62.93, 70.09)	31.69 (30.44, 32.99)	56.57 (55.28, 57.91)
40–49 years	38.71 (37.19, 40.29)	67.14 (63.97, 70.46)	31.17 (30.36, 31.99)	58.50 (57.37, 59.65)
50–59 years	34.85 (33.68, 36.06)	52.04 (49.92, 54.24)	30.71 (30.15, 31.27)	55.15 (54.35, 55.95)
60–69 years	30.12 (29.08, 31.20)	45.94 (44.24, 47.72)	28.73 (28.25, 29.22)	47.40 (46.74, 48.08)
70–79 years	36.42 (34.97, 37.93)	49.94 (48.08, 51.88)	30.56 (30.07, 31.07)	44.41 (43.79, 45.04)
Without comorbidity				
18–39 years	45.52 (42.55, 48.70)	62.03 (58.06, 66.26)	28.91 (27.20, 30.72)	49.81 (48.08, 51.58)
40–49 years	36.33 (34.56, 38.19)	63.64 (59.69, 67.85)	29.66 (28.55, 30.82)	57.36 (55.73, 59.04)
50–59 years	32.32 (30.91, 33.79)	50.85 (48.12, 53.74)	29.22 (28.43, 30.03)	54.13 (52.97, 55.30)
60–69 years	27.94 (26.56, 29.39)	44.81 (42.38, 47.38)	26.67 (25.96, 27.40)	45.48 (44.49, 46.49)
70–79 years	33.13 (30.91, 35.49)	46.87 (43.89, 50.06)	29.11 (28.29, 29.95)	42.91 (41.93, 43.92)
With comorbidity				
18–39 years	53.34 (48.19, 59.03)	77.32 (70.44, 84.88)	34.27 (32.48, 36.17)	63.31 (61.38, 65.30)
40–49 years	43.94 (41.08, 46.98)	72.42 (67.27, 77.96)	32.61 (31.47, 33.80)	59.50 (57.94, 61.10)
50–59 years	39.14 (37.13, 41.27)	53.64 (50.39, 57.11)	32.05 (31.26, 32.84)	56.03 (54.94, 57.14)
60–69 years	32.55 (30.97, 34.19)	46.96 (44.60, 49.44)	30.22 (29.58, 30.88)	48.87 (47.97, 49.79)
70–79 years	38.42 (36.55, 40.39)	51.64 (49.29, 54.10)	31.36 (30.74, 31.99)	45.34 (44.54, 46.15)

Data are unadjusted rates PTPY with 95% CI

### Risk of depression in YOD compared with usual-onset type 2 diabetes patients

**Fig. 2:**
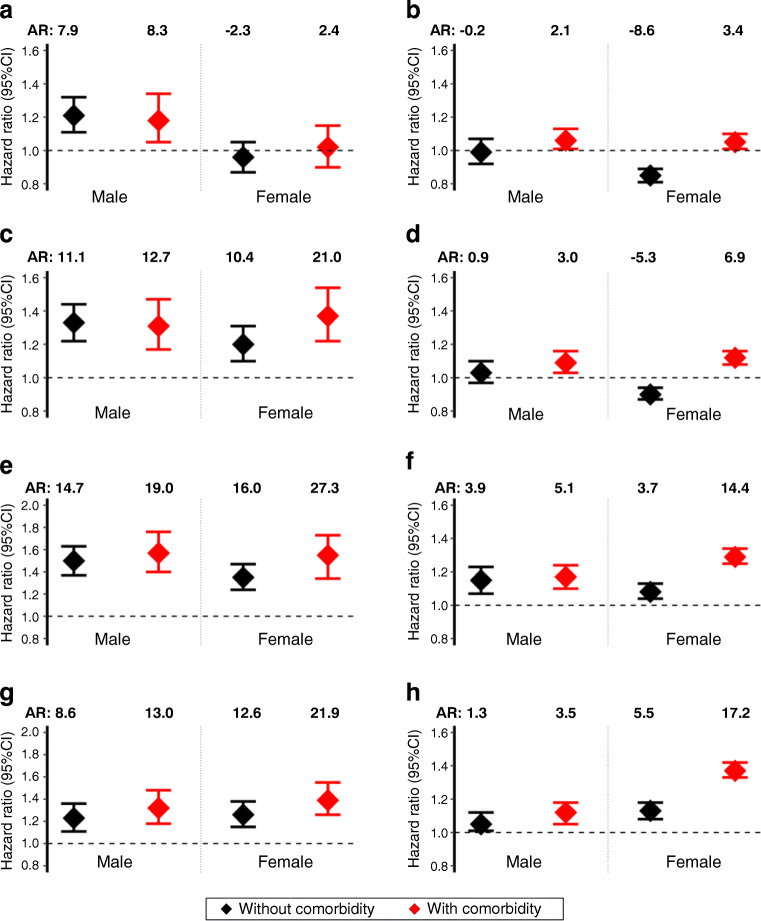
Adjusted HR (95% CI) of developing depression in patients diagnosed with type 2 diabetes at age 18–39 years compared with those diagnosed at 40–49 years (**a**, **b**), those aged 18–39 years compared with those aged 50–59 years (**c**, **d**), those aged 18–39 years compared with those aged 60–69 years (**e**, **f**), and those aged 18–39 years compared with those aged 70–79 years (**g**, **h**) in the UK cohort (**a**, **c**, **e**, **g**) and the USA cohort (**b**, **d**, **f**, **h**). Comparisons were made for patients with baseline comorbidities (red symbols) and without baseline comorbidities (black symbols). AR represents the absolute risk (additional depression cases PTPY). The horizontal dashed lines represent an HR of 1, indicating no difference between young- and usual-onset diabetes

The adjusted HR (95% CI) and AR for depression in people aged 18–39 years compared with older age groups, separately in men and women by baseline comorbidity status, are presented in Fig. 2 and ESM Table 5. In the UK, compared with people aged ≥50 years, the youngest men had a 23–57% significantly higher risk of developing depression (AR 8.6–19.0 cases PTPY) and the youngest women had a 20–55% significantly higher risk of developing depression (AR 10.4–27.3 cases PTPY); the results were, similar for those with and those without comorbidity at type 2 diabetes diagnosis. In the USA, an increased depression risk in those aged 18–39 years was observed compared with those aged ≥60 years with or without comorbidities: the risk increased in men by 5–17% (AR 1.3–5.1 cases PTPY) and that in women increased by 8–37% (AR 3.7–17.2) (all *p* <0.01).

## Discussion

This longitudinal study, based on two cohorts of approximately 1.4 million people with incident type 2 diabetes from population-representative EMRs from two different healthcare systems, offers new insight into the depression risk dynamics in YOD and usual-onset type 2 diabetes patients. The primary findings are: (1) a significant increasing trend in the prevalence of depression at the time of type 2 diabetes diagnosis, which is similar across all age groups in both countries, and (2) that men and women with YOD (aged <40 years at time of diagnosis) have a significantly higher risk of developing depression compared with those who developed type 2 diabetes at ≥50 years, with the risk being similar irrespective of cardiometabolic comorbidities at the time of type 2 diabetes diagnosis.

We observed a significant increasing trend in the prevalence of depression and AMI at the time of type 2 diabetes diagnosis, which was similar across all age groups in men and women in both countries. Although the prevalence of a cardiometabolic comorbidity at the time of type 2 diabetes diagnosis has been found to be highest among people aged ≥60 years at the time of type 2 diabetes diagnosis in both countries [2, 5, 29], we observed that the depression prevalence is similar across all age groups.

Although our depression prevalence estimates at the time of type 2 diabetes diagnosis for the UK are higher than in the USA (UK 35%; USA 25%), the estimates are comparable with those obtained in earlier studies from Europe and the USA in established type 2 diabetes populations: 23% (CI 18, 28%) in Europe [3] and 25% (CI 23, 28%) in the USA [11].

A novel finding of our study is the significantly higher risk of developing depression in patients with YOD compared with patients with usual-onset type 2 diabetes, with the risk estimates being similar for people with and without comorbidities at the time of type 2 diabetes diagnosis. In the UK, these risk estimates were similar for people with and without comorbidities in both men and women across all increasing age groups. A similar trend was observed in US men. However, the patterns of risk among US women were different by baseline comorbidity status, particularly in the comparisons with age groups 40–49 and 50–59 years (Fig. 2b and d). The observed difference could be due to unmeasured mediation effects, which requires further investigation. This clearly indicates the mental health implications of developing diabetes at an early age irrespective of underlying comorbidities. While the pathophysiology of depression in people with type 2 diabetes has been discussed [1, 6], several factors, including a higher burden of risk factors including obesity in YOD, may partially explain the higher risk of developing depression in patients with YOD compared with usual-onset type 2 diabetes. A recent USA CEMR data-based study reported a similar mediation effect of depression across all age groups after diagnosis of type 2 diabetes on the increased cardiovascular risk [5]. However, to further evaluate the observed higher depression risk in YOD irrespective of comorbidity status at type 2 diabetes diagnosis, future studies evaluating the mediation effects of the time-varying cardiometabolic diseases and risk factors before and after type 2 diabetes diagnosis on depression risk in different age groups, sex and ethnicity would be of great importance.

As observed in this study, the prevalence and incidence of depression in people with type 2 diabetes are significantly higher among women in both countries, with the rate of increase in the prevalence of depression among women also being consistently higher across all age groups, compared with men. While recent studies using these UK or USA EMRs have reported the overall prevalence of depression and other comorbidities at onset of type 2 diabetes [2, 5, 15, 29], we are not aware of any study that explored the population-level trend in depression prevalence at the time of type 2 diabetes diagnosis across age groups and sex [11]. Understanding the recent changing dynamics of cardiometabolic comorbidity and depression in patients with YOD and usual-onset type 2 diabetes is of paramount importance for proactive engagement of primary care teams in population-level mental health management and healthcare cost reduction. The 2004–2011 Medical Expenditure Panel Survey from the USA showed that the average medical cost for patients with diabetes and symptomatic depression was more than double compared with people with diabetes and no depression [30].

Despite the sociodemographic and healthcare system differences between the UK and the USA, all age groups experienced statistically significant increases in comorbid depression during the study period (Fig. 1). A plausible reason for the 2–9% annual increase in the rates of comorbid depression is an increased awareness and likelihood of diagnosis in primary/ambulatory care, as more research and education about the association between diabetes and depression emerges [3, 11]. In addition, better record-keeping as a result of the transition to EMRs would have resulted in an increased likelihood of capturing secondary medical diagnoses including depression. This is reflected in the overall temporal prevalence of depression for the USA, with a significant annual percentage change observed from 2009 onwards (ESM Fig. 2).

Proactive management of comorbid depression in terms of timely screening, early diagnosis and pharmacotherapeutic treatment may lead to improved glycaemic and other risk factor control in people with diabetes, delayed onset of complications and lower healthcare-associated costs. Petrak et al [31] recommend treating depression first, as the response to medications is usually seen within weeks after initiation of antidepressant treatment, while improvement in the glycaemic control requires several months. Given the increasing rate of comorbidities and the varying dynamics of different sociodemographic populations, innovative approaches to identify subgroups of patients for proactive management will be beneficial. More research is required to understand the dynamics and patterns of management of patients with depression to improve outcomes for patients with diabetes and other comorbidities including depression. In addition, given the complexity of the roles of comorbidities in the interplay of diabetes and depression, detailed evaluation of the bidirectional association between these conditions in different ethnicities, age groups and sex is crucial [32].

The main strength of our study is the simultaneous evaluation of longitudinal data from two nationally representative primary/ambulatory care EMRs from different healthcare systems in the UK and the USA over a period of 11 years. Compared with cross-sectional surveys that primarily capture self-reported symptomatology at a single point in time, EMR data provides information on a wealth of comorbidities based on reliable clinical diagnoses. In addition, patient data in EMRs can be linked to longitudinal patient-level medical and clinical records; making it possible to explore temporal associations between risk factors and disease outcomes, including depression [5].

There are several unavoidable limitations in outcome studies based on EMRs. The under-reporting of depression is a common problem globally. Mis-coding of conditions is a common limitation when using EMRs. However, we used clinically guided machine learning-based methods to identify people with type 2 diabetes and depression. There is bias in recording of depression over time, and difficulties identifying people who have been receiving prescriptions for antidepressants that are meant to be used for treating depression only (in the absence of clinical codes for depression). The increasing prevalence of comorbid depression may reflect an increase in the actual incidence of depression but may also be due to several other factors, including physician awareness and diagnosis or documentation practices. Also, the availability of socioeconomic, smoking status and ethnicity data was not consistent in the EMRs from the UK and the USA. Other limitations include unavoidable indication bias and residual confounding, which are common problems in any EMR-based outcome studies, together with a lack of data on physical activity, the nature of insurance, education, income, other cultural drivers, missing HbA_1c_ results and lack of reliable data on competing risks such as death. While mortality is an important competing risk in the context of outcome studies with real-world longitudinal EMRs, we were unable to perform any sensitivity analysis accounting for competing risks due to death, as the CEMR database does not provide death data and deaths are poorly recorded in the THIN database. Furthermore, while obtaining reliable information on medication adherence is a common problem in all clinical studies, detailed validation studies of these EMRs suggested a high level of agreement between EMR prescription data and pharmacy claims data, especially for chronic diseases [33].

In conclusion, the prevalence and incidence of depression among people with incident type 2 diabetes in the UK and the USA are rapidly increasing across all age groups, particularly in those with YOD. Men and women with YOD have a significantly higher risk of developing depression compared with those with usual-onset type 2 diabetes, with the risk being similar in people with and without comorbidities at type 2 diabetes diagnosis. It is recommended that clinicians screen regularly for depression in people with incident type 2 diabetes, particularly among those who are <50 years old, irrespective of their cardiometabolic comorbidity status.

## Supplementary information

ESM (PDF 241 kb)

## Data Availability

The patient-level EMRs used for this study comes with non-sharing license agreements with the data providers. However, the metadata can be provided upon request.

## Abbreviations

AMI: Any mental illness
AR: Absolute risk
CEMR: Centricity Electronic Medical Records
EMR: Electronic Medical Records
PTPY: Per 1000 patient-years
THIN: The Health Improvement Network
YOD: Young-onset type 2 diabetes
