# Supplementary material for: Temporal trends in the prevalence and incidence of depression and the interplay of comorbidities in patients with young- and usual-onset type 2 diabetes from the USA and the UK
Source: Diabetologia. 2022 Sep 5;65(12):2066–77. doi: 10.1007/s00125-022-05764-9 (PMC9630215; doi:10.1007/s00125-022-05764-9)

Electronic Supplementary Materials (ESM)

Table of contents

ESM Table 1: Antidepressant generic ingredients commonly prescribed for mental illnesses in UK…………..2

ESM Table 2: Antidepressant generic (brand) drugs commonly prescribed for mental illnesses in US………………………………………………………………. …………………………………………..3

ESM Table 3. Distribution of deprivation score by age groups for UK cohort …………………………………4

ESM Table 4. Distribution of ethnicity by age groups for US cohort……………………………………………4

ESM Table 5. The adjusted effects of young-onset vs. usual-onset diabetes with and without baseline comorbidities on the risk of depression onset in individuals with incident T2DM from the UK and US…………5

ESM Fig 1: Flow chart for the selection of study cohorts…………………………………………………………6

ESM Fig 2: Observed and modelled trend in the prevalence (a) and incidence rates per 1000 person-years (b) of depression among incident type 2 diabetes patients from UK and US. The dots and triangles represent observed rates while the lines are the estimates from the JPR model. APC=Annual percent change………………………7

TABLES

ESM Table 1. Antidepressant generic ingredients commonly prescribed for mental illnesses in UK.

Drug class	Generic name	Indications	Age groups
Tricyclic antidepressants (TCAs)	Amitriptyline, Clomipramine, Dosulepin (dothiepin), Doxepin, Imipramine, Lofepramine, Nortriptyline, Trimipramine	Depressive illness, neuropathic pain, phobic and obsessional states, adjunctive treatment of cataplexy associated with narcolepsy	Above 18
Monoamine -oxidase inhibitors (MAOIs)	Phenelzine, Isocarboxazid, Tranylcypromine.	Depressive illness	above 18 (not for child)
Reversible MAOIs (RIMAs)	Moclobemide	Depressive illness, social anxiety disorder	above 18 (not for child)
Selective serotonin reuptake inhibitors (SSRIs)	Citalopram, Escitalopram, Fluoxetine, Fluvoxamine, Paroxetine, Sertraline, Vortioxetine	Depressive illness, panic disorder, generalised anxiety disorder, obsessive-compulsive disorder, social anxiety disorder, Bulimia nervosa, post-traumatic stress disorder, generalised anxiety disorder	Above 18
Serotonin-noradrenaline reuptake inhibitors (SNRIs)	Duloxetine, Venlafaxine	Major depressive disorder, generalised anxiety disorder	Above 18
Other antidepressants	Mianserin, Trazodone, Agomelatine, L-tryptophan, Mirtazapine, Reboxetine	Depressive illness, anxiety, major depression, treatment resistant depression, generalised anxiety disorder	Above 18

ESM Table 2. Antidepressant generic (brand) drugs commonly prescribed for mental illnesses US.

Drug class	Generic name	Brand name	Indications
Tricyclic antidepressants (TCAs)	Amitriptyline	ALAVIL(Elavil), ENTRAVIL, LIMBITROL, TRIAVIL, VANATRIP, ETRAFON	Major depressive disorder (MDD), anxiety disorders, and less commonly attention deficit hyperactivity disorder (ADHD) and bipolar disorder,
	Amoxapine	ASENDIN	Anxiety, depression
	Clomipramine	ANAFRANIL	Depression, anxiety, panic disorder
	Doxepin	SILENOR, SINEQUAN	Insomnia (low dose of doxepin), depression, anxiety
	Trimipramine, Desipramine, Imipramine	SURMONTIL, NORPRAMIN, TOFRANIL,	Depression
	Nortriptyline	PAMELOR, AVENTYL	Depression, ADHD
	Protriptyline	VIVACTIL	Depression, ADHD
Monoamine -oxidase inhibitors (MAOIs)	Isocarboxazid	MARPLAN	Depression
	Phenelzine	NARDIL	Depression
	Tranylcypromine	PARNATE	Depression
Selective serotonin reuptake inhibitors (SSRIs)	Fluoxetine	Prozac, SYMBYAX, SARAFEM, SELFEMRA	MDD, obsessive compulsive disorder (OCD), bulimia nervosa, panic disorder, and premenstrual dysphoric disorder (PMDD), depression associated with bipolar, anxiety, Anorexia Nervosa, Posttraumatic Stress Syndrome (PTSD), Binge Eating Disorder
	Sertraline	ZOLOFT	Depression, OCD, panic disorder, PTSD, social anxiety disorder, and PMDD
	Paroxetine	PAXIL, PEXEVA	Depression, OCD, panic attacks, anxiety disorders, PTSD, and PMDD
	Citalopram	CELEXA, CIPRAMIL	Depression, anxiety, OCD, panic disorder, PTSD, and PMDD
	Escitalopram	LEXAPRO, CIPRALEX	Anxiety in adults and MDD
	Fluvoxamine	LUVOX	OCD, depression
	Vortioxetine	BRINTELLIX, TRINTELLIX	Major depressive disorder
Serotonin-noradrenaline reuptake inhibitors (SNRIs)	Venlafaxine, Desvenlafaxine	EFFEXOR, PRISTIQ, KHEDEZLA	Anxiety, depression
	Duloxetine	CYMBALTA, IRENKA	Depression, anxiety
	Levomilnacipran	FETZIMA	Depression
Other antidepressants	Buproprion	BUDEPRION¸ BUPROBAN, WELLBUTRIN, APLENZIN, APPBUTAMONE, FORFIVO, APLENZIN	Depression
	Trazodone	OLEPTRO, DESYREL	Depression
	Mirtazapine	REMERON	Depression
	Nefazodone	SERZONE	Depression
	Maprotiline	LUDIOMIL	Depression, anxiety
	Vilazodone	VIIBRYD, TIANEPTINE	Depression, anxiety

ESM Table 3. Distribution of deprivation score by age groups for UK cohort

Townsend	18-39 yrs	40-49 yrs	50-59 yrs	60-69 yrs	70-79 yrs	Overall
1 (least deprived)	2022 (11)	5278 (14)	10,223 (17)	12,905 (19)	9826 (20)	49,254 (17)
2	2255 (12)	5439 (15)	10,012 (17)	12,753 (19)	10,035 (20)	40,494 (18)
3	3521 (19)	6994 (19)	11,249 (19)	12,742 (19)	9330 (19)	43,836 (19)
4	4203 (22)	7542 (20)	10,908 (18)	11,518 (17)	8568 (17)	42,739 (19)
5 (most deprived)	3724 (20)	6619 (18)	8855 (15)	8557 (13)	5779 (12)	33,534 (15)
**Missing**	3084 (16)	5285 (14)	7858 (13)	8098 (12)	5750 (12)	30,075 (13)

ESM Table 4. Distribution of ethnicity by age groups for US cohort.

Ethnicity	18-39 yrs	40-49 yrs	50-59 yrs	60-69 yrs	70-79 yrs	Overall
Black	15,115 (18)	23,503 (16)	41,375 (15)	36,210 (11)	23,953 (8)	140,156 (12)
White	51,825 (61)	93,168 (63)	185,906 (66)	226,851 (71)	222,865 (72)	780,615 (68)
Others	3418 (4)	5284 (4)	8529 (3)	9115 (3)	7316 (2)	33,662 (3)
**Missing**	14,493 (17)	24,998 (17)	46,147 (16)	48,938 (15)	54,113 (18)	188,689 (17)

ESM Table 5. The adjusted effects of young-onset vs. usual-onset diabetes with and without baseline comorbidities on the risk of depression onset in individuals with incident T2DM from the UK and US.

Effects	18-39 vs. 40-49 yrs UK	18-39 vs. 40-49 yrs US	18-39 vs. 50-59 yrs UK	18-39 vs. 50-59 yrs US	18-39 vs. 60-69 yrs UK	18-39 vs. 60-69 yrs US	18-39 vs. 70-79 yrs UK	18-39 vs. 70-79 yrs US
**Male**								
**AR (95% CI)**								
without comorbidity	7.97 (4.40, 11.5)	-0.24 (-2.33, 1.86)	11.1 (7.67, 14.5)	0.94 (-0.99, 2.87)	14.7 (11.3, 18.1)	3.87 (1.96, 5.78)	8.63 (4.75, 12.5)	1.27 (0.90, 3.23)
with comorbidity	8.33 (2.16, 14.5)	2.06 (0.96, 4.24)	12.7 (6.88, 18.5)	3.04 (1.04, 5.04)	19.0 (13.3, 24.7)	5.11 (3.15, 7.07)	13.0 (7.20, 18.8)	3.50 (1.54, 5.46)
**HR (95% CI)**								
without comorbidity	1.21 (1.11, 1.32)	0.99 (0.92, 1.07)	1.33 (1.22, 1.44)	1.03 (0.97, 1.10)	1.50 (1.37, 1.63)	1.15 (1.07, 1.23)	1.23 (1.11, 1.36)	1.05 (1.01, 1.12)
with comorbidity	1.18 (1.05, 1.34)	1.06 (1.01, 1.13)	1.31 (1.17, 1.47)	1.09 (1.03, 1.16)	1.57 (1.40, 1.76)	1.17 (1.10, 1.24)	1.32 (1.18, 1.48)	1.12 (1.05, 1.18)
**Female**								
**AR (95% CI)**								
without comorbidity	-2.28 (-8.06, 3.50)	-8.63 (-11.0, -6.22)	10.4 (5.38, 15.4)	-5.25 (-7.35, -3.15)	16.0 (11.1, 20.9)	3.70 (1.68, 5.72)	12.6 (7.34, 17.8)	5.45 (3.43, 7.47)
with comorbidity	2.36 (-6.64, 11.4)	3.36 (0.83, 5.89)	21.0 (13.0, 29.0)	6.88 (4.63, 9.13)	27.3 (19.7, 34.9)	14.4 (12.2, 16.6)	21.9 (14.3, 29.5)	17.2 (15.1, 19.3)
**HR (95% CI)**								
without comorbidity	0.96 (0.87, 1.05)	0.85 (0.81, 0.89)	1.20 (1.10, 1.31)	0.90 (0.87, 0.94)	1.35 (1.24, 1.47)	1.08 (1.04, 1.13)	1.26 (1.15, 1.38)	1.13 (1.08, 1.18)
with comorbidity	1.02 (0.90, 1.15)	1.05 (1.01, 1.10)	1.37 (1.22, 1.54)	1.12 (1.08, 1.16)	1.55 (1.34, 1.73)	1.29 (1.25, 1.34)	1.39 (1.26, 1.55)	1.37 (1.33, 1.42)

AR: AR represents the absolute risk (additional depression cases per 1000 person-years) between comparator groups. AR was estimated from Aalen's additive survival model.

HR: adjusted Hazard Ratio. HR was estimated from Weibull survival model.

In UK, the model was adjusted for smoking status, deprivation status and hypertension, In US, the model was adjusted for smoking status, race/ethnicity and hypertension. All analyses were conducted separately in people with and without comorbidity at T2DM diagnosis.

FIGURES

ESM Fig 1: Flow chart for the selection of study cohorts

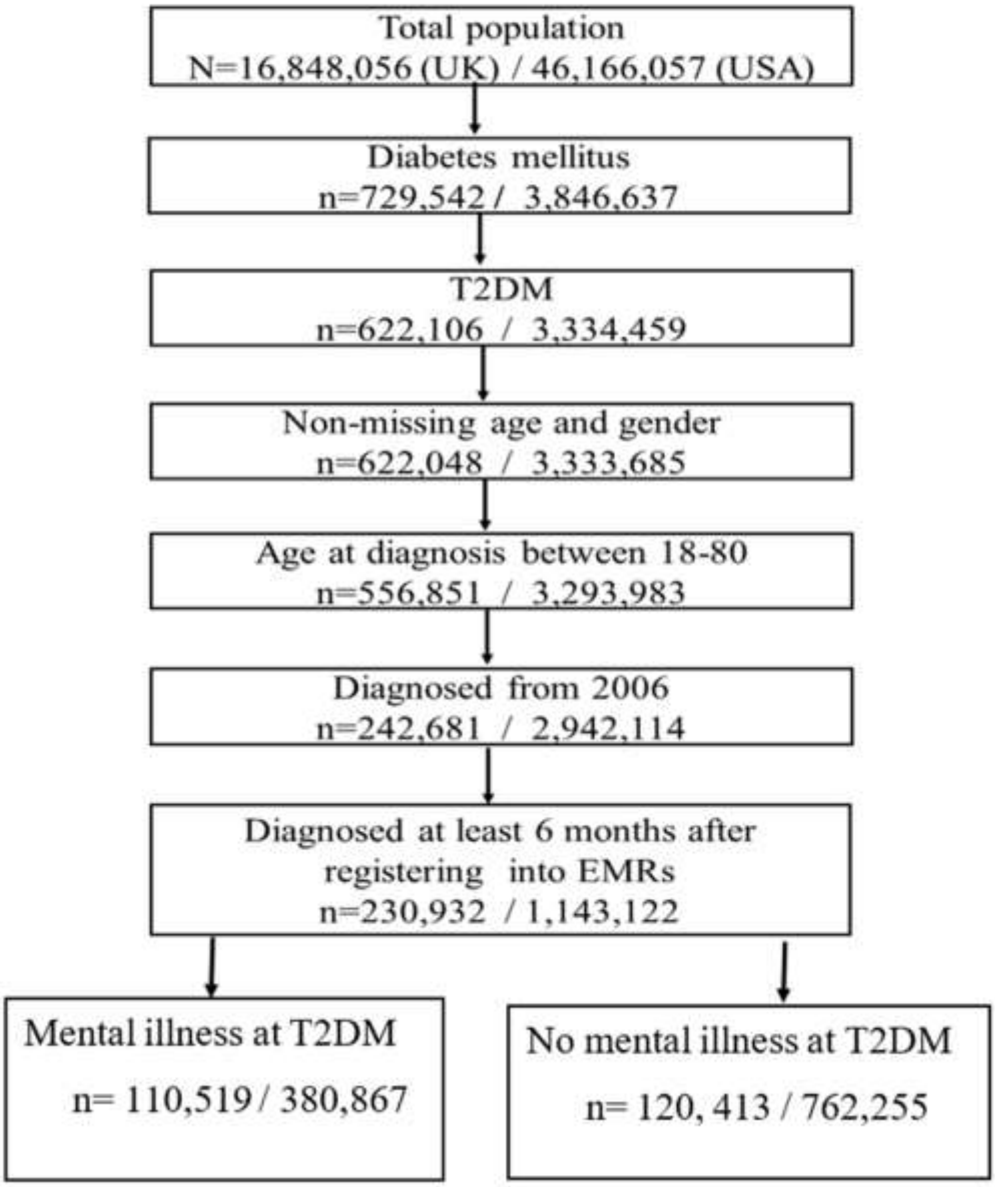

ESM Fig 2: Observed and modelled trend in the prevalence (**a**) and incidence rates per 1000 person-years (**b**) of depression among incident type 2 diabetes patients from UK and US. The dots and triangles represent observed rates while the lines are the estimates from the JPR model. APC=Annual percent change.

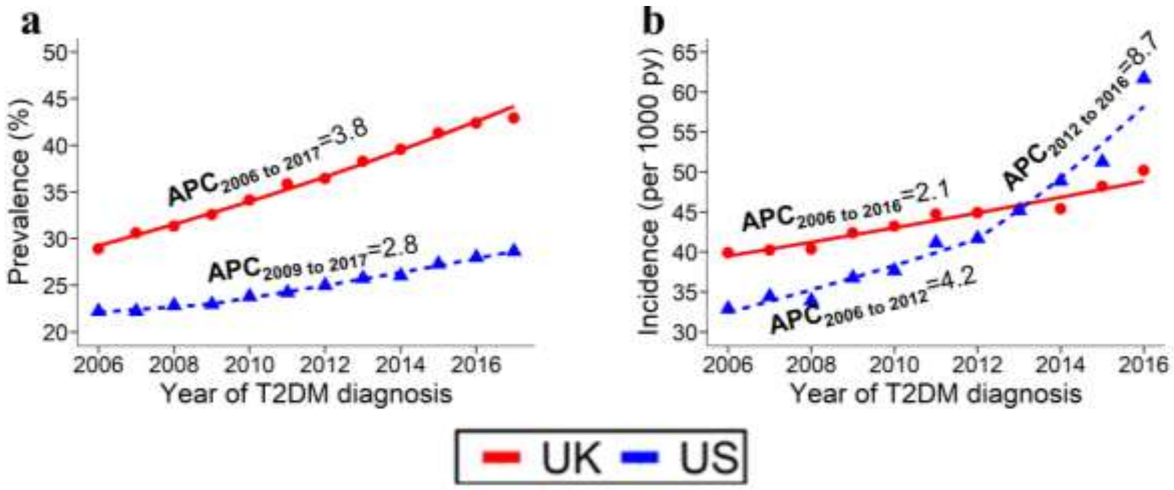

Supplement: Supplementary file 1 — (PDF 241 kb) [file 125_2022_5764_MOESM1_ESM.pdf]
